# Editorial: Improving exercise testing methods and interpretation in human health and diseases

**DOI:** 10.3389/fphys.2023.1188429

**Published:** 2023-04-04

**Authors:** Mathieu Gruet, Martin Behrens, Leonardo A Peyré-Tartaruga

**Affiliations:** ^1^ IAPS Laboratory, University of Toulon, Toulon, France; ^2^ Department of Sport Science, Institute III, Otto von Guericke University Magdeburg, Magdeburg, Germany; ^3^ Programa de Pós-Graduação Em Ciências Pneumológicas, Hospital de Clínicas de Porto, Alegre/Universidade Federal Do Rio Grande Do Sul, Porto Alegre, RS, Brazil; ^4^ LaBiodin Biodynamics Laboratory, Universidade Federal Do Rio Grande Do Sul, Porto Alegre, RS, Brazil; ^5^ Programa de Pós-Graduação em Ciências do Movimento Humano, Universidade Federal Do Rio Grande Do Sul, Porto Alegre, RS, Brazil

**Keywords:** physical fitness, six minute walk test, cardiopulmonary exercise testing, muscle function, chronic diseases

## Introduction

Exercise testing is a valuable diagnostic tool that is pivotal to successful exercise prescription. Several new testing modalities and outcomes have emerged in the last decades, broadening the applications of exercise testing and indications for regular assessments. However, there is still much to do to improve exercise testing methods, implementation, interpretation, and significance in specific clinical contexts. The present research topic followed a multimodal approach to exercise testing without any restrictions regarding populations, reflecting the editors’ aspiration to highlight the complementary of different exercise tools and outcomes for a wide range of clinical applications. This research topic includes 17 peer-reviewed articles covering various exercise methods (from local muscle testing to maximal whole-body exercise), populations and diseases, including healthy children and adults with various fitness levels and several chronic diseases and pathological conditions: cystic fibrosis, chronic obstructive pulmonary disease (COPD), lung cancer resection candidates, coronary artery disease, individuals with left ventricular assist devices, chronic kidney disease, obesity and bariatric surgery candidates.

## Incremental exercise tests: Useful clinical information

Incremental exercise tests with gas exchange measurements objectively assess the integrative functioning of the respiratory, cardiovascular, and muscular systems. These tests are useful to detect physiological adaptations and abnormalities, providing avenues for optimizing exercise prescription. Youxiang et al. found different exercise metabolic responses during incremental treadmill tests between obese children and adolescents with no insulin resistance and those with insulin resistance, with for instance, a lower maximal fat oxidation intensity for the latter, which should be considered when designing exercise interventions targeting fat loss. Tomlinson et al. retrospectively analysed cardiopulmonary exercise testing (CPET) of people with cystic fibrosis of differing glycemic status. They found that people with cystic fibrosis-related diabetes (CFRD) had reduced peak oxygen uptake (V̇O_2peak_) compared to non-CFRD counterparts, which was linked to poorer lung function. They also found that lung function and aerobic capacity differed in direction and magnitude of longitudinal changes, suggesting that V̇O_2peak_ should be considered an independent clinical marker in people with CF with varying glycemic status.

CPET-derived outcomes also have a good prognostic value in various clinical populations. For instance, V̇O_2peak_ can predict mortality and hospitalization in cardiac and respiratory diseases (Arena et al., 2004; Hebestreit et al., 2019). However, such predictive capacity remains to be established in some disorders and specific subgroups of patients, notably for predicting postoperative complications after cardiopulmonary surgery. For instance, while V̇O_2_ thresholds have proven utility in stratifying postoperative risk following lung cancer resection, they were primarily derived from patients who underwent thoracotomy (Brunelli et al., 2009). Chouinard et al. found that V̇O_2peak_ was also an independent predictor of postoperative morbidity and mortality after minimally invasive video-assisted thoracoscopic surgery for lung cancer resection. However, its ability to discriminate patients with or without adverse outcomes was limited.

CPET is also an important tool for athletes. For example, it might be helpful to determine how the performance of the cardiorespiratory system in athletes could be affected during periods of training reduction or cessation (e.g., off-season, near-competition, post-injury/surgery period). However, performing a maximal CPET might not always be advisable in these circumstances. With this framework in mind, Oyarzo-Aravena et al. sought to identify the determinants of the cardiorespiratory optimal point, corresponding to the lowest minute ventilation to V̇O_2_ ratio obtained during a CPET in endurance athletes. Using principal component analysis, they found that this submaximal parameter was closely related to the second ventilatory threshold and V̇O_2peak_, supporting its utility to track physiological performance in the aforementioned circumstances, where a submaximal assessment might be preferred.

Predicting V̇O_2peak_ might also be of value in athletes for better interpretation of the test or when indirect calorimetry is unavailable. However, some predictive equations might be misleading if they are utilized in populations that differ from those selected to develop the prediction model. For instance, some physiological impairments may be masked in athletes if their performances are carelessly compared to predictive values derived from non-athletic populations. Jurov et al. compared the V̇O_2peak_ measured in 580 competitive cyclists to V̇O_2peak_ estimated from four predictive equations and found that the FRIEND (Fitness Registry and the Importance of Exercise National Database) equation was the most suitable for predicting V̇O_2peak_ in this athletic population.

## Incremental exercise tests: New procedures and outcomes for intensity distribution

CPET-derived outcomes like ventilatory or lactate thresholds are also relevant to set training intensities. However, the need for special equipment and operators has stimulated research to develop new methods to detect exercise intensity zones. Rogers and Gronwald discussed in a narrative review the utility of the short-term scaling exponent alpha1 of Detrended Fluctuation Analysis (DFA a1), an HRV index based on fractal correlation properties, to delineate exercise intensity domains. Discrete numerical values of this indicator have shown great associations with physiological thresholds in both athletic and clinical populations. Another promising application of DFA a1 is the management of internal load for specific exercise modalities, which have proven efficacy in improving health markers in several populations but for which assessing intensity distribution is not straightforward [e.g., eccentric cycling (Barreto et al., 2021)].

Several studies have evaluated the ability of various versions of the Talk Test to discriminate exercise intensities, intending to make exercise prescription even more simple and accessible. For instance, as the ability to vocalize is becoming more difficult with increasing ventilatory requirements, some studies found a good approximation of the ventilatory threshold from these Talk Tests (Persinger et al., 2004). To provide a more standardized form of the Talk Test, Mahmod et al., developed the time-controlled monosyllabic Talk Test. They found that this version was more suited to detect the intensity of light, moderate, and vigorous exercise intensity, as compared to self-paced Counting Talk Test, offering new avenues in the field of exercise prescription.

## Six-minute walk test

While the previous sections supported the clinical significance of CPET, the value of alternative exercise tests should not be discarded. Field exercise tests can be used in settings where access to CPET is limited and may provide complementary information and be better suited for regular assessments. The 6-min walk test (6MWT) is among the most popular field exercise tests. While it provides only limited information on the causes of exercise limitation, the 6MWT remains widely used as a simple low-cost alternative for measuring functional exercise capacity in a wide range of clinical populations. This point is illustrated by two systematic reviews published in this research topic that benefited from the fact that this test is often selected as a clinical trial endpoint to discuss the efficacy of exercise interventions in specific clinical contexts based on changes in 6MWT performance. Song et al. used changes in 6MWT as a surrogate of training efficacy in hemodialysis patients and found that the combination of aerobic plus resistance exercise was the most effective intervention to improve 6MWT in this population. In the context of bariatric surgery, Jabbour et al. discussed the importance of enhancing the preoperative fitness levels of patients to improve postoperative outcomes. In this context, this review highlights the clinical significance of the 6MWT, which is the most used exercise test and that has proven to be highly feasible and sensitive to detect functional improvements in bariatric surgery candidates.

It is also important to interpret the performance of the 6MWT for exercise counselling, and performance is often compared to a predicted performance from a reference equation. However, some equations obtained from a healthy population may not be adapted for interpreting performance in specific clinical populations. Lenasi et al. demonstrated that the widely used Enright-Sherill equation leaded to a prediction that was 52 m lower than the actual performance of people with stable coronary artery disease, pointing out the importance of developing populations-specific prediction equations.

Although the 6MWT is a simple and well-tolerated test in several clinical populations, it still has the inconvenience of requiring a quiet corridor of at least 30 m, which might be a limitation for its implementation in some hospital settings. The 6-min step test (6MST), a portable test that only requires a stepper, can thus be an interesting alternative. The clinimetric properties of the 6MST have been established in various populations, including stable people with COPD (Borel et al., 2010). Ribeiro et al. extended its use to hospitalized COPD patients with an acute exacerbation by confirming its concurrent validity, offering a new simple alternative to guide and monitor therapeutic strategies in these fragile patients.

## Local peripheral muscle testing

Muscle exercise testing encompasses various components, i.e., strength, power, endurance and fatigability. Altered muscle function may negatively impact various activities of daily living and seems predictive for various health issues, such as sports injuries or falls. Muscle exercise testing allows to identify specific muscle abnormalities in a given population and having reliable and accessible tests and outcomes will help to guide therapeutic interventions and assess their effectiveness. For instance, using isometric and isokinetic testing of several muscle groups, Gobbo et al. reported large impairments in muscle strength in people with left ventricular assist devices, which were not correlated with V̇O_2peak_. While these results offer a rationale for implementing resistance training modalities in this population, they also suggest that increasing muscle strength may not be sufficient to translate into improved aerobic endurance performance. Muscle testing may also allow to detect functional differences between populations and shed light on the underlying mechanisms. For instance, the meta-analysis conducted by Souron et al. showed that the differences in muscle fatigability and endurance between healthy children and adults were dependent on the exercise testing modality (i.e., isometric vs. dynamic exercise tests).

Vertical jump testing is another form of muscle testing, which offers simple markers of neuromuscular function that are relevant to daily life activities. Jump tests can be performed safely even in old individuals (Buehring et al., 2015) and jump height can be reliably estimated with a force plate, which is considered the gold-standard method. Nevertheless, as this latter is not always easily accessible, Gruber et al. sought to assess the validity and reliability of counter movement jump height using a sport watch (Polar Vantage V2). Their positive results support the use of this technology to reliably monitor jump height outside the lab, fostering the implementation of jump testing in clinical settings.

Muscle testing may also be useful in the context of injury prevention, for instance, in alpine skiers by detecting potential muscle imbalances derived from the hamstrings-to-quadriceps strength ratio (Spörri et al., 2017). However, such outcomes are often not sufficient and it might be useful to complement these functional measures with muscle architecture assessments. This is supported by the findings of Fitze et al., who found that average anatomical cross-sectional area of the biceps femoris long head measured by ultrasound was associated with the occurrence of traumatic lower extremity injuries in youth skiers. The cost and low portability of high-end ultrasonography devices is however a limitation to their implementation in sports and clinical settings. Ritsche et al. demonstrated that lower limb muscle architecture measurements like muscle thickness could be reliably assessed with handheld portable ultrasound system with good agreement with a high-end laboratory device, fostering the possibility of developing new screening methods and algorithms in the context of injury prevention based on the combination of affordable muscle architecture and functional assessments.

## Assessment of exercise-induced gastrointestinal perturbations

Exercise testing can also be helpful in identifying markers or predictors of syndromes associated with strenuous/prolonged exercises and/or performed under environmental stress. For instance, various hypoxic exercise tests have been developed to identify physiological predictors of severe high-altitude illness (Richalet et al., 2012). Some exercise modalities can also be used to study exercise-induced gastrointestinal and systemic disturbances (Costa et al., 2022). Young et al. demonstrated that a 2 h high-intensity interval exercise was sufficient for inducing gastrointestinal and systemic disturbances, with variable reliability (from poor to excellent) of several usual biomarkers. These data support the need to assess a cluster of biomarkers and interpret them collectively to determine the incidence and severity of exercise-induced gastrointestinal syndrome.

## Conclusion

The collective publications in this research topic support exercise testing as a versatile tool providing relevant information in virtually all populations, irrespective of age, disease, and severity. Such studies are pivotal in fostering the implementation of exercise testing in clinical practice and guiding the choice among all the existing testing procedures.
